# Short-term forecasting of the COVID-19 outbreak in India

**DOI:** 10.1093/inthealth/ihab031

**Published:** 2021-06-05

**Authors:** Sherry Mangla, Ashok Kumar Pathak, Mohd Arshad, Ubydul Haque

**Affiliations:** Department of Mathematics and Statistics, Central University of Punjab, Bathinda, Punjab, India 151401; Department of Mathematics and Statistics, Central University of Punjab, Bathinda, Punjab, India 151401; Department of Mathematics, Indian Institute of Technology Indore, Simrol, Indore, India 453552; Department of Statistics and Operations Research, Aligarh Muslim University, Aligarh, India 202002; Department of Biostatistics and Epidemiology, University of North Texas Health Science Center, Fort Worth, TX, USA

**Keywords:** ARIMA, COVID-19, forecasting, logistic growth model

## Abstract

As the outbreak of coronavirus disease 2019 (COVID-19) is rapidly spreading in different parts of India, a reliable forecast for the cumulative confirmed cases and the number of deaths can be helpful for policymakers in making the decisions for utilizing available resources in the country. Recently, various mathematical models have been used to predict the outbreak of COVID-19 worldwide and also in India. In this article we use exponential, logistic, Gompertz growth and autoregressive integrated moving average (ARIMA) models to predict the spread of COVID-19 in India after the announcement of various unlock phases. The mean absolute percentage error and root mean square error comparative measures were used to check the goodness-of-fit of the growth models and Akaike information criterion for ARIMA model selection. Using COVID-19 pandemic data up to 20 December 2020 from India and its five most affected states (Maharashtra, Karnataka, Andhra Pradesh, Tamil Nadu and Kerala), we report 15-days-ahead forecasts for cumulative confirmed cases and the number of deaths. Based on available data, we found that the ARIMA model is the best-fitting model for COVID-19 cases in India and its most affected states.

## Introduction

The coronavirus disease 2019 (COVID-19) pandemic is spreading around the world.^1^ Human-to-human transmission has been confirmed and, worldwide, measures have been taken to mitigate the virus’ spread.^2^ This pandemic has placed an unprecedented burden on the global economy, healthcare and globalization through its effects on travel, events cancellation, employment, the food chain, academia and healthcare capacity.^3^ According to the Worldometer website (https://www.worldometers.info/coronavirus/), as of 20 December 2020, there were 77.75 million cases globally and around 10 million confirmed cases in India. The first case of COVID-19 was reported in India on 30 January 2020. As the number of COVID-19 cases increased significantly since the first case was reported, the government of India imposed a complete lockdown on 25 March 2020. Due to the unavailability of drugs to cure COVID-19, most countries are implementing stringent laws for isolation and quarantine of infected people. India is the second most populous country in the world and contains 18% of the world’s population as of 2019.^4^ Because of this, it is important to predict the cumulative number of infected cases and associated deaths in India.

In the current situation of the COVID-19 pandemic, decision making and strategy planning activities rely on accurate forecasts of the disease. Numerous researchers have used various modelling techniques for forecasting COVID-19 cases in different countries, including short-term forecasting such as the autoregressive integrated moving average (ARIMA) and Holt's exponential smoothing in India,^5^ the simple mean-field model and susceptible–infected–recovered–deaths model,^6^ the Gompertz model, the logistic model and the Bertalanffy model^7^ in Italy, China and France. In the literature, researchers have used the Gompertz model to predict the growth of tumours,^8^ bacteria^9^ and birds,^10^ whereas the logistic growth model has been used for 29 provinces of China and around the world to model the outbreak of COVID-19^11^ and to forecast the worldwide spread of COVID-19.^12^ Similarly, the exponential growth model was employed to model coal production in Nigeria^13^ and population growth^14^ and the ARIMA model has been used to forecast the final size and spread of COVID-19 in Italy^15^ and the cumulative confirmed cases of COVID-19 for Mainland China, Italy, South Korea, Iran and Thailand.^16^

In this study, the cumulative number of infected cases and the total number of deaths in India after the announcement different unlock phases are predicted using four different models: exponential, Gompertz, logistic growth and ARIMA. Mean absolute percentage error (MAPE) and root mean square error (RMSE) values were used to measure the goodness-of-fit of the model. The model with the smallest MAPE and RMSE values is considered best. Nejadettehad et al.^17^ used MAPE and RMSE metrics to compare the performance of the recurrent neural network, gated recurrent unit and long- and short-term memory neural network in short-term traffic flow prediction. Qian et al.^18^ used identical metrics to compare the artificial neural network model (i.e. Elman recurrent neural network) and the classical time series model (i.e. seasonal autoregressive integrated moving average) to estimate and forecast traffic death cases in China. A similar performance evaluation procedure was adopted by Huang and Hao^19^ and Zhou et al.^20^

India ranked second on the pandemic vulnerability index and the morbidity and mortality due to COVID-19 is spreading rapidly in India. The proposed models will provide a reliable forecast for outbreaks at the national and state level to implement interventions to curb the pandemic.^3^

## Methods

### Data collection

The daily reported cumulative number of infected cases and deaths from 30 January to 20 December 2020 was collected from the COVID19-India API website (https://api.covid19india.org/documentation/csv/). State-level data for the total number of confirmed cases were collected from 14 March to 20 December 2020. Irregularities in the daily reported cases affect the time series and hence the cumulative number of cases were analysed, which provides more stable and reliable results.

### Exponential growth model

One of the most common applications of exponential functions involves growth and decay models. In a range of physical processes, exponential growth and decay make an appearance. Exponential functions are widespread in nature, from population growth to radioactive decay. In infectious disease modelling, when a function *CC_t_* continues to expand at a rate *r*>0, then *CC_t_* has the form

(1) \begin{equation*}C{C_t} = {I_0}\ {e^{rt}},\end{equation*}

where *CC_t_* is the cumulative number of infected cases at time *t. I*_0_ is the initial number of cumulative infected cases and *r* is the growth rate.

### Logistic growth model

Logistics equations were introduced by the seminal work of Pierre-Francois Verhulst in 1844–1845.^21^ The logistic growth model illustrates that population growth is confined by carrying capacity and the growth rate gets smaller and smaller as population size approaches the carrying capacity. Hence the logistic growth model assumes that the growth rate decreases linearly with size until it equals zero at the carrying capacity. Logistic growth models are mainly used in epidemiology, biology and environmental sciences. It is important to investigate the risk factor of a serious disease and to estimate the possibility of the outbreak of disease based on the risk factors. The growth and transmission law of epidemiology can be approximately estimated by using a logistic growth curve:

(2) \begin{equation*}C{C_t} = \frac{{{M_c}}}{{1 + {e^{a - b\left( {t - {t_0}} \right)}}}}\ ,\end{equation*}

where *CC_t_* is the cumulative number of confirmed cases at time *t, M_c_* is the predicted maximum of confirmed cases, *a* and *b* are fitting coefficients and *t*_0_ is the time when the first case was reported.

### Gompertz model

The Gompertz model is widely used and a well-known technique to model the population growth and has many applications in biology, epidemiology and environmental science. This model was introduced by Gompertz^22^ as an animal population growth model to describe the extinction law of the population. Also, the Gompertz model is a particular case of the Richards model. The development of epidemic growth is equivalent to the growth of the population. In this article the Gompertz model was used to determine the cumulative number of COVID-19 infected cases in India. The mathematical form is:

(3) \begin{equation*}C{C_t} = {M_c}\ {e^{ - a{e^{ - b\left( {t - {t_0}} \right)}}}},\end{equation*}

where *CC_t_* is the cumulative number of confirmed cases at time *t, M_c_* is the predicted maximum of confirmed cases, *a* and *b* are fitting coefficients and *t*_0_ is the time when the first case was reported.

### ARIMA model

ARIMA models are classical techniques of time series forecasting introduced by Box and Jenkins.^23^ ARIMA (*p, d, q*) models are a combination of autoregressive AR(*p*) and moving average MA(*q*) models, where *p* represents the order of autoregressive terms, *d* is the degree of difference and *q* is the order of the moving average. The ARIMA (*p, d, q*) model is given by:

(4) \begin{equation*}{y_t} = {{\rm{a}}_0} + {{\rm{a}}_1}{y_{t - 1}} + \ldots + {a_p}{y_{t - p}} + {b_1}{e_{t - 1}} + \ldots + {b_q}{e_{t - q}} + {e_t},\end{equation*}

where *y_t_* is the time series under consideration, *e_t_* is the error at time *t* and *a* and *b* and are coefficients.

### Confidence interval (CI) estimation

Estimation of parameters leads to a specific point estimate. In practice, point estimates frequently vary from the parameter's actual value. In order to tackle this, the *t*-statistic was considered to construct the CIs in this article for different model estimates. The CI approach for the mean (μ) was utilized as:

$$\begin{equation*}{\bar{X}_n} \pm {t_{1 - \alpha /2}}S/\sqrt n ,\end{equation*}$$

where ${t_{1 - \alpha /2}}$ specifies the Student's *t*-distribution with *n*−1 degree of freedom and *S* is the sample standard deviation.

## Results and Discussion

**Figure 1.**
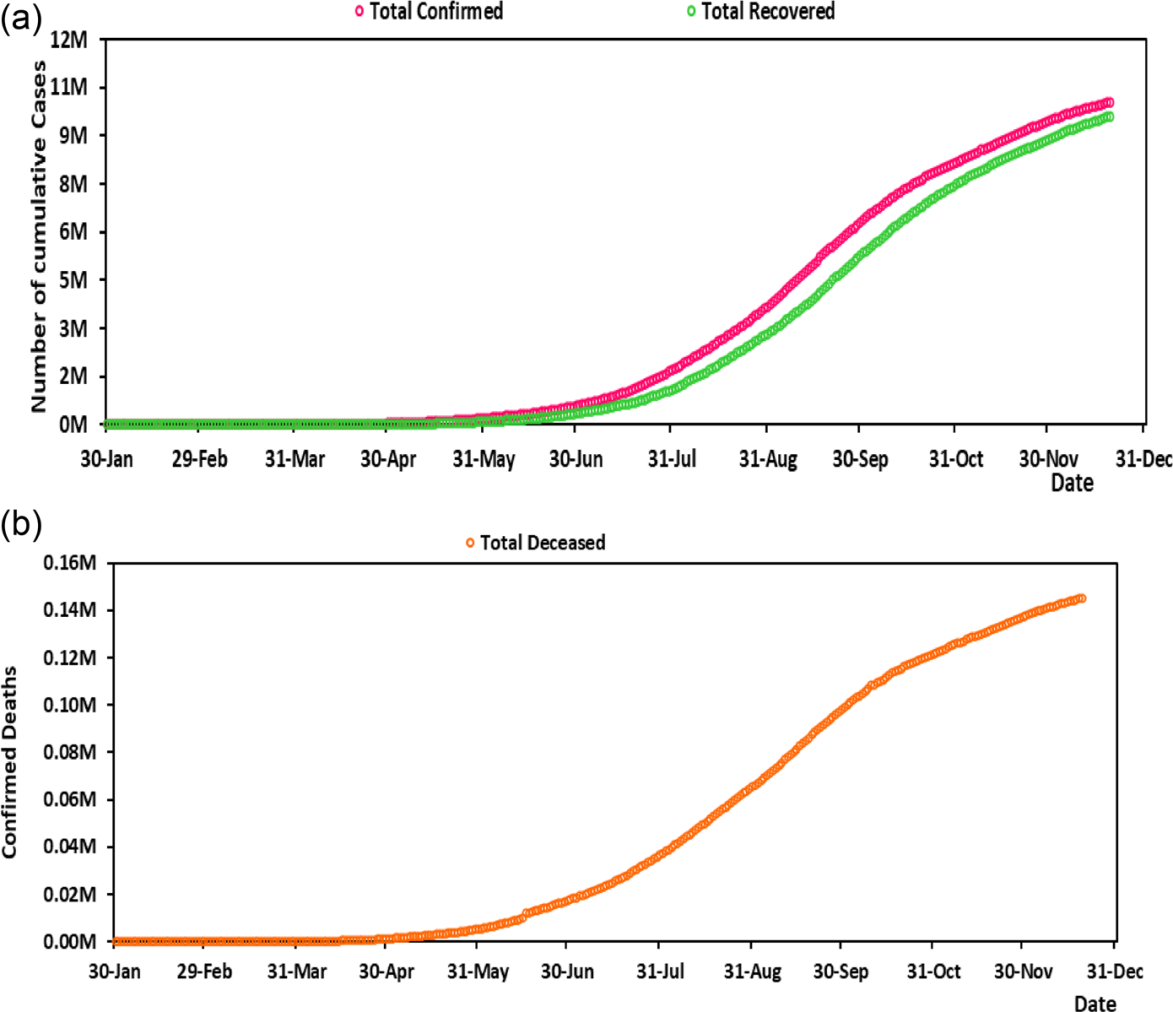
(a) Cumulative number of confirmed cases and recovered cases (in millions) of COVID-19 in India through 20 December 2020. (b) Cumulative number of confirmed deaths (in millions) due to COVID-19 in India through 20 December 2020.

**Figure 2.**
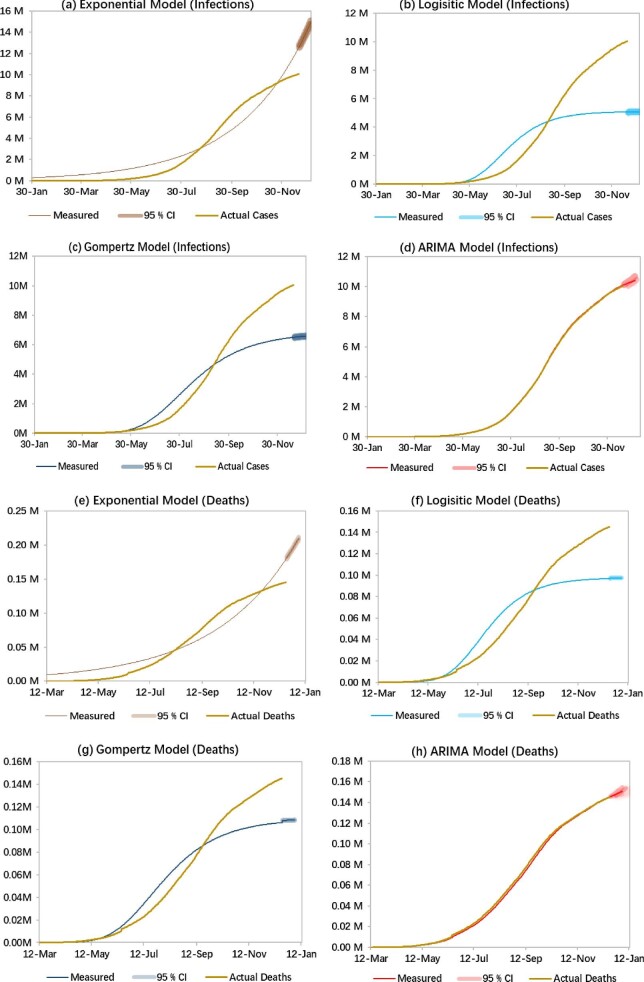
Expected cumulative number of cases (in millions) with 95% confidence limits from 21 December 2020 to 4 January 2021 using (a) the exponential growth model, (b) the logistic growth model; (c) the Gompertz model and (b) the ARIMA model. Expected cumulative deaths (in millions) with 95% confidence limits from 21 December 2020 to 4 January 2021 using (e) the exponential growth model, (f) the logistic growth model, (g) the Gompertz model and (h) the ARIMA model.

The 15-days-ahead forecast of COVID-19 for India was generated using four different methods, the exponential growth model, logistic growth model, Gompertz model and ARIMA model, from 21 December 2020 to 4 January 2021. The cumulative number of confirmed cases and recovered cases in India from 30 January to 20 December 2020 is presented in Figure 1a and the cumulative number of deaths until 20 December 2020 is presented in Figure 1b. The 15-days-ahead forecast for the cumulative cases and deaths from each model is shown in Figure 2. Tables 1 and 2 show the expected number of cumulative confirmed cases of the four models with 95% CIs and Tables 3 and 4 show the expected number of deaths using the four models with their 95% CIs. Also, to improve the forecast, we fed the truncated time series to generate a 15-days-ahead forecast from 21 December 2020 to 4 January 2021. R version 4.0.2 (R Foundation for Statistical Computing, Vienna, Austria) was used for this analysis.

**Table 1.** Results of the 15-days-ahead forecasts for the cumulative number of infected cases (in millions) of COVID-19 in India using the exponential growth and logistic growth models.

	Exponential growth model			Logistic growth model		
Date	Lower CI	Measured	Upper CI	Lower CI	Measured	Upper CI
21 December 2020	12.56	12.69	12.82	5.05	5.07	5.10
22 December 2020	12.71	12.84	12.97	5.05	5.08	5.10
23 December 2020	12.86	12.99	13.12	5.05	5.08	5.10
24 December 2020	13.01	13.14	13.28	5.06	5.08	5.10
25 December 2020	13.16	13.30	13.44	5.06	5.08	5.10
26 December 2020	13.32	13.46	13.59	5.06	5.08	5.10
27 December 2020	13.47	13.61	13.76	5.06	5.08	5.10
28 December 2020	13.63	13.78	13.92	5.06	5.08	5.10
29 December 2020	13.79	13.94	14.08	5.06	5.08	5.10
30 December 2020	13.96	14.10	14.25	5.06	5.08	5.10
31 December 2020	14.12	14.27	14.42	5.06	5.08	5.10
1 January 2021	14.29	14.44	14.59	5.06	5.08	5.11
2 January 2021	14.46	14.61	14.76	5.06	5.08	5.11
3 January 2021	14.63	14.78	14.94	5.06	5.08	5.11
4 January 2021	14.80	14.96	15.12	5.06	5.09	5.11

**Table 2.** Results of the 15-days-ahead forecasts for the cumulative number of infected cases (in millions) of COVID-19 in India using the Gompertz and ARIMA models.

	Gompertz growth model			ARIMA model		
Date	Lower CI	Measured	Upper CI	Lower CI	Measured	Upper CI
21 December 2020	6.48	6.51	6.53	10.12	10.13	10.14
22 December 2020	6.49	6.51	6.54	10.14	10.15	10.18
23 December 2020	6.49	6.52	6.54	10.15	10.17	10.21
24 December 2020	6.50	6.52	6.55	10.16	10.19	10.24
25 December 2020	6.51	6.53	6.55	10.17	10.21	10.28
26 December 2020	6.51	6.54	6.56	10.19	10.23	10.32
27 December 2020	6.52	6.54	6.56	10.20	10.25	10.35
28 December 2020	6.52	6.55	6.57	10.21	10.27	10.39
29 December 2020	6.53	6.55	6.58	10.22	10.29	10.43
30 December 2020	6.53	6.56	6.58	10.22	10.31	10.47
31 December 2020	6.54	6.56	6.59	10.23	10.33	10.51
1 January 2021	6.54	6.57	6.59	10.24	10.35	10.55
2 January 2021	6.55	6.57	6.59	10.25	10.36	10.60
3 January 2021	6.55	6.58	6.60	10.25	10.38	10.65
4 January 2021	6.56	6.58	6.60	10.26	10.40	10.70

**Table 3.** Results of the 15-days-ahead forecasts for the cumulative number of deaths (in millions) due to COVID-19 in India using the exponential growth and logistic growth models.

	Exponential growth model			Logistic growth model		
Date	Lower CI	Measured	Upper CI	Lower CI	Measured	Upper CI
21 December 2020	0.180	0.181	0.183	0.097	0.097	0.098
22 December 2020	0.181	0.183	0.185	0.097	0.097	0.098
23 December 2020	0.183	0.185	0.187	0.097	0.097	0.098
24 December 2020	0.185	0.187	0.189	0.097	0.097	0.098
25 December 2020	0.187	0.189	0.191	0.097	0.097	0.098
26 December 2020	0.189	0.191	0.193	0.097	0.097	0.098
27 December 2020	0.191	0.193	0.195	0.097	0.097	0.098
28 December 2020	0.193	0.195	0.197	0.097	0.097	0.098
29 December 2020	0.195	0.197	0.199	0.097	0.097	0.098
30 December 2020	0.197	0.199	0.201	0.097	0.097	0.098
31 December 2020	0.199	0.201	0.203	0.097	0.097	0.098
1 January 2021	0.201	0.203	0.205	0.097	0.097	0.098
2 January 2021	0.203	0.205	0.207	0.097	0.097	0.098
3 January 2021	0.206	0.208	0.210	0.097	0.098	0.098
4 January 2021	0.208	0.210	0.212	0.097	0.098	0.098

**Table 4.** Results of the 15-days-ahead forecasts for the cumulative number of deaths (in millions) due to COVID-19 in India using the Gompertz and ARIMA models.

	Gompertz growth model			ARIMA model		
Date	Lower CI	Measured	Upper CI	Lower CI	Measured	Upper CI
21 December 2020	0.108	0.108	0.108	0.146	0.146	0.146
22 December 2020	0.108	0.108	0.108	0.146	0.146	0.147
23 December 2020	0.108	0.108	0.108	0.146	0.147	0.147
24 December 2020	0.108	0.108	0.108	0.146	0.147	0.148
25 December 2020	0.108	0.108	0.108	0.146	0.147	0.148
26 December 2020	0.108	0.108	0.108	0.147	0.148	0.149
27 December 2020	0.108	0.108	0.108	0.147	0.148	0.149
28 December 2020	0.108	0.108	0.109	0.147	0.148	0.150
29 December 2020	0.108	0.108	0.109	0.147	0.149	0.151
30 December 2020	0.108	0.108	0.109	0.147	0.149	0.151
31 December 2020	0.108	0.108	0.109	0.148	0.149	0.152
1 January 2021	0.108	0.108	0.109	0.148	0.150	0.153
2 January 2021	0.108	0.108	0.109	0.148	0.150	0.153
3 January 2021	0.108	0.108	0.109	0.148	0.151	0.154
4 January 2021	0.108	0.108	0.109	0.148	0.151	0.155

### Exponential growth model

The exponential growth model was fitted to data from 30 January to 20 December 2020 for the cumulative number of infected cases and deaths. The results of the forecast from this model are shown in Tables 1 and 3. The findings of the exponential model show that there will be around 14.96 million (95% CI 14.80 to 15.12) cumulative cases and 0.210 million (95% CI 0.208 to 0.212) cumulative deaths on 4 January 2021 in India.

### Logistic growth model

Using the logistic growth model, we expect 5.09 million (95% CI 5.06 to 5.11) cumulative infected cases and 0.098 million (95% CI 0.097 to 0.098) cumulative deaths in India on 4 January 2021, as shown in Tables 1 and 3. Log transformation was used for variance stabilization to perform this method over the cumulative number of cases from 30 January to 20 December 2020 and deaths from 12 March to 20 December 2020.

### Gompertz model

Using the Gompertz model, it is expected that there will be 6.58 million (95% CI 6.56 to 6.60) cumulative infected cases and 0.108 million (95% CI 0.108 to 0.109) deaths in India on 4 January 2021. Tables 2 and 4 present the results of the forecasts for the model.

The non-linear least square method was used to estimate the parameters for the three models: exponential growth model, logistic growth model and Gompertz model. The R^2^ values for the exponential, logistic and Gompertz models for cumulative confirmed cases were 0.934, 0.974 and 0.988 and for the total number of deaths were 0.926, 0.961 and 0.976, respectively. Hence we conclude that the Gompertz model provides a better fit among the three models for the considered dataset.

### ARIMA model

ARIMA models were fitted to daily infected cases from 30 January to 20 December 2020 to generate 15-days-ahead forecasts from 21 December 2020 to 4 January 2021. To check the stationarity of the time series, an augmented Dickey–Fuller (ADF) test and Kwiatkowski–Phillips–Schmidt–Shin (KPSS) test were performed, and the best model was selected based on the smallest Akaike information criterion (AIC) value. Since the time series was not stationary, one difference was taken to achieve stationarity. Meanwhile, two was added to every observation of the time series to avoid the situation of an undefined MAPE. ARIMA (1, 2, 5) was selected as the best model for cumulative infected cases, with an AIC value of −118.78, which was the smallest among the models. We used logarithmic transformation to stabilize the variance. Residual analysis was performed to ensure satisfactory performance of the model. The ARIMA (1, 2, 5) model passed the Ljung–Box test, with a p-value of 0.34. According to the ARIMA (1, 2, 5) model, it is expected that there will be about 10.40 million (95% CI 10.26 to 10.70) cumulative infected cases on 4 January 2021 (see Table 2).

**Figure 3.**
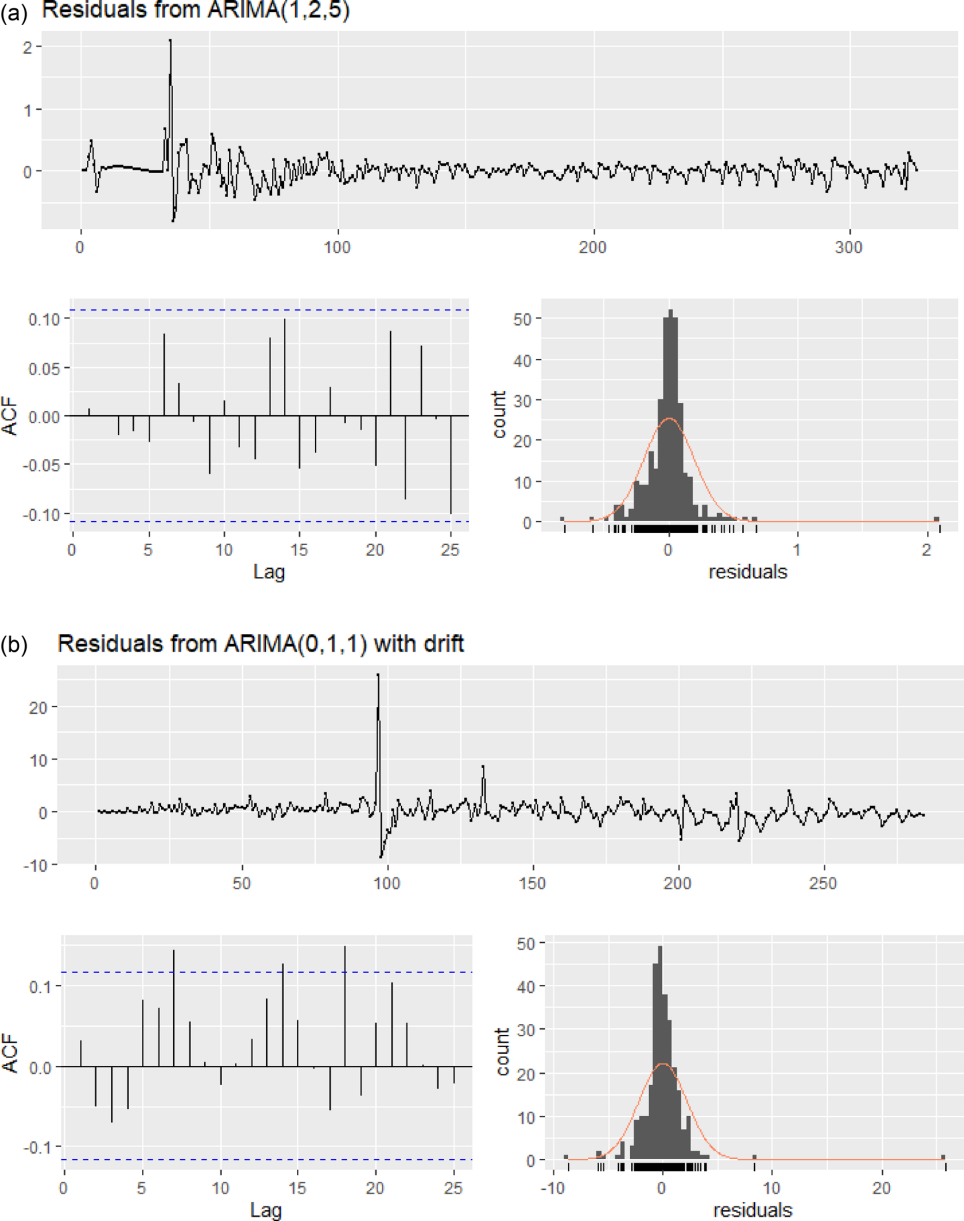
(a) Residuals of the ARIMA (1, 2, 5) model and (b) the ARIMA (0, 1, 1) model.

**Figure 4.**
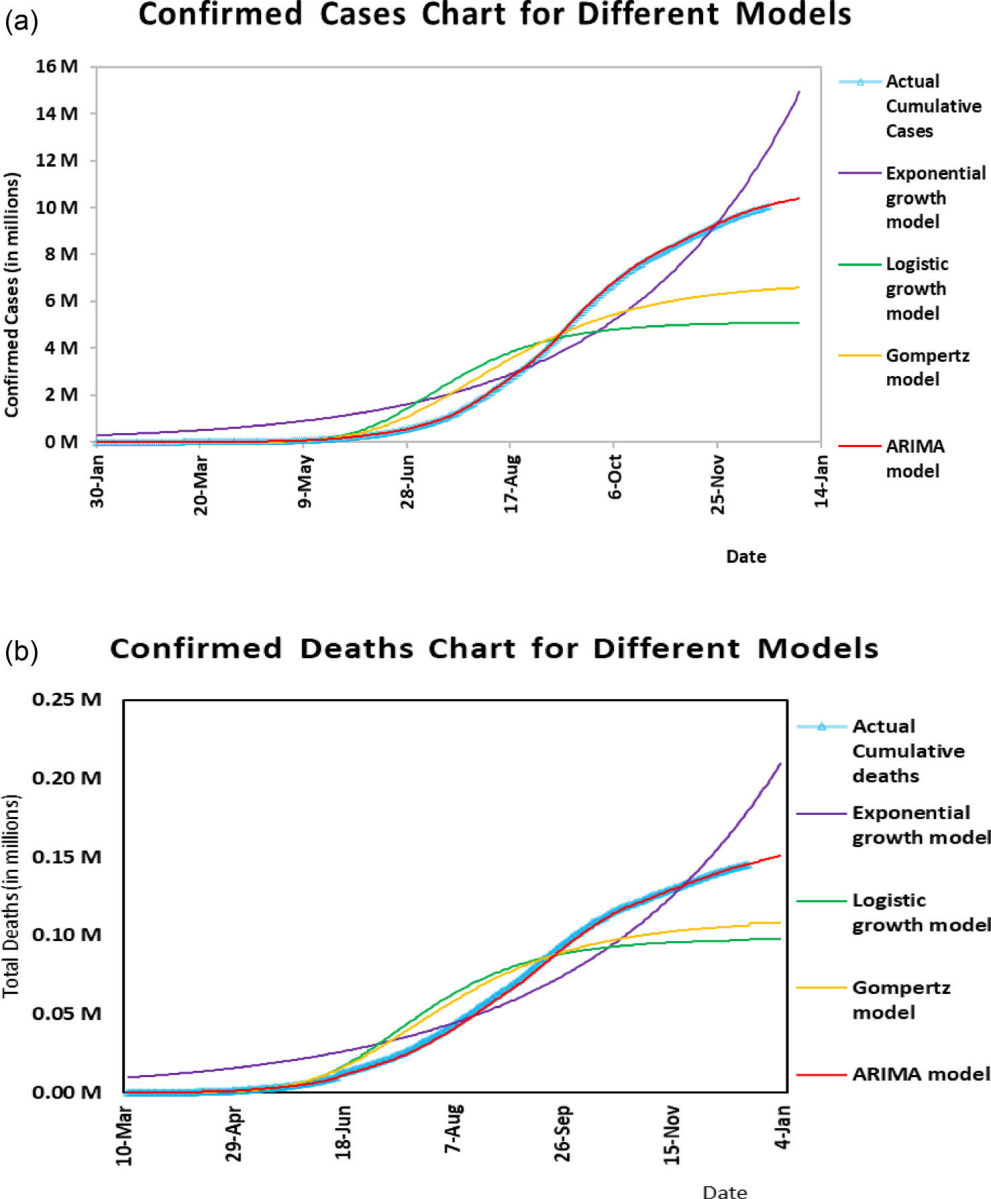
(a) Comparison plot of all four models for the cumulative number of infected cases (in millions) of COVID-19 in India along with the actual reported cases and (b) for the cumulative number of deaths (in millions) due to COVID-19 in India along with the actual reported deaths.

Figure 3a represents the residuals of the ARIMA (1, 2, 5) model. Residuals are randomly scattered around a zero mean with constant variance and follow an approximately normal distribution. Also, there is no serial correlation in residuals. While predicting the cumulative number of deaths using the ARIMA model, a similar methodology was employed and ARIMA (0, 1, 1) was selected as the best model, with a corresponding AIC value of 1256.51. ARIMA (0, 1, 1) passed the Ljung–Box test with a p-value of 0.12. According to this model, there will be around 0.151 million (95% CI 0.148, 0.155) expected deaths on 4 January 2021 (see Table 4). The residual plot for ARIMA (0, 1, 1) is given in Figure 3b. Fourth-root transformation was used to stabilize the variance of residuals. A comparison of all four models is presented in Figure 4. To estimate the parameters of the ARIMA model, a conditional sum of squares followed by the maximum likelihood (CSS-ML) estimation method was used. First, a minimum conditional sum-of-squares was used to find the starting values, then the maximum likelihood estimation method was applied.

Results of comparative performance using MAPE and RMSE between the models are presented in Table 5. In Figure 2, it is seen that of the four models, ARIMA fitted values nearly coincide with the actual reported values (infections and deaths) from 30 January to 20 December 2020, defining a better fit of the forecast using the ARIMA model. Thus the ARIMA model was employed for forecasting the cumulative number of infected cases at the regional level.

**Table 5.** Comparative performance results of the exponential, logistic, Gompertz and ARIMA models for infected and deceased cases.

	Infected cases		Deceased cases	
Model	MAPE	RMSE	MAPE	RMSE
Exponential	13 540.28	915 734	199.71	14 141
Logistic	0.87	1 936 295	0.58	19 631
Gompertz	0.52	1 351 553	0.40	15 634
ARIMA	0.03	0.20	2.99	0.22

### Forecasting at the regional level

**Figure 5.**
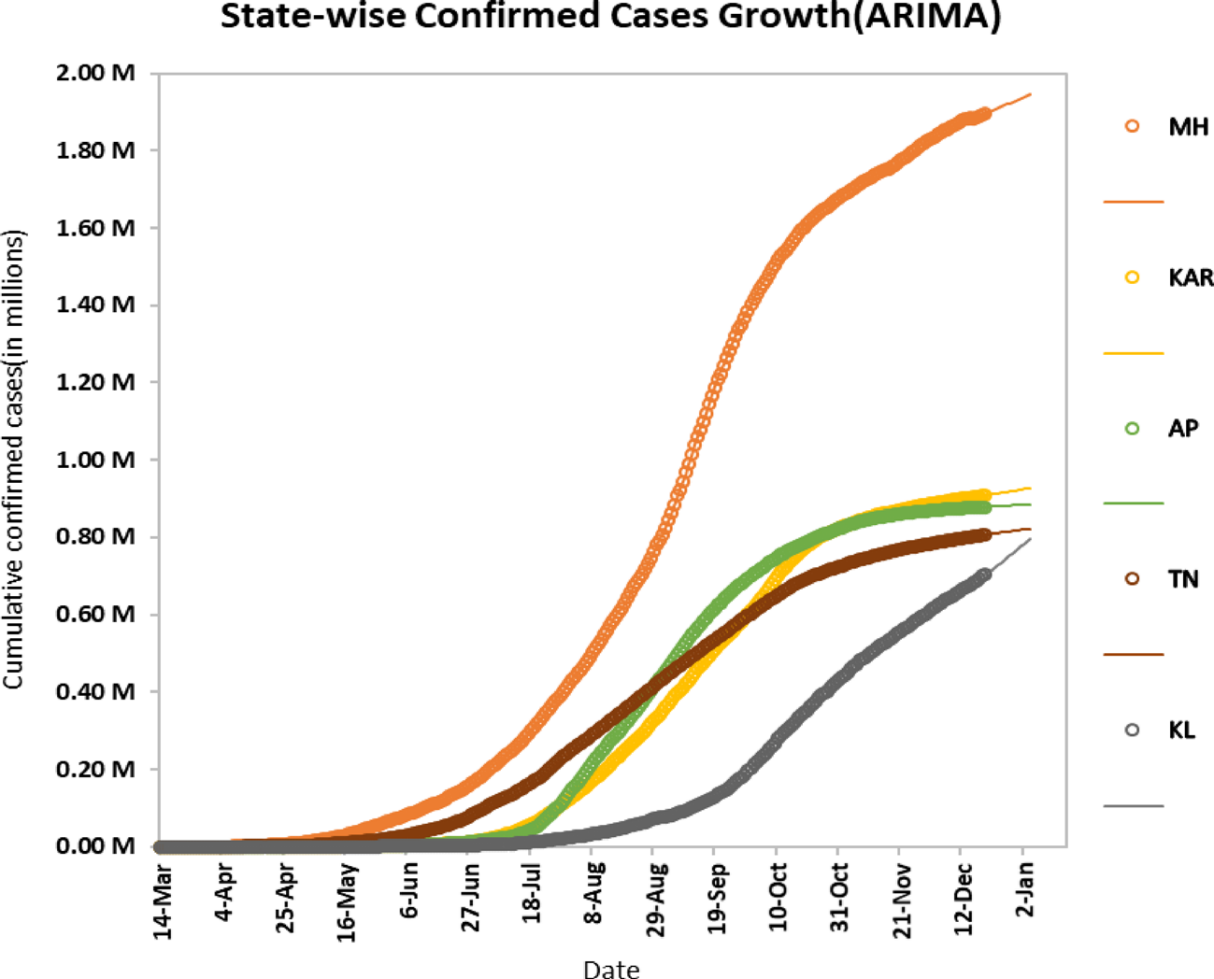
Plots of the 15-days-ahead forecast for the cumulative number of confirmed cases (in millions) in Maharashtra (MH), Andhra Pradesh (AP), Karnataka (KA), Tamil Nadu (TN) and Uttar Pradesh (UP) from 21 December 2020 to 4 January 2021.

In this study, five states (Maharashtra, Karnataka, Andhra Pradesh, Tamil Nadu and Kerala) were included for forecasting at the regional level. The time series of daily infected cases from 14 March to 20 December 2020 were used to provide the 15-days-ahead forecast. We found that Maharashtra will be the most affected state, with approximately 1.94 million cumulative cases, and Kerala will be least affected among these states, approximately with 0.80 million cumulative cases. ARIMA models were found to be suitable at the regional level and the results of the 15-days-ahead forecasts are given in Table 6. A graphical representation of the forecast from 21 December 2020 to 4 January 2021 for Maharashtra, Karnataka, Andhra Pradesh, Tamil Nadu and Kerala is shown in Figure 5 (where the dots represent actual cases until 20 December 2020 and the lines represent predictions from the ARIMA model through 4 January 2021). Maharashtra [ARIMA (3,2,3) with drift], Karnataka [ARIMA (5, 2, 5) with drift], Andhra Pradesh [ARIMA (0, 2, 2) with drift], Tamil Nadu [ARIMA (1, 2, 2) with drift] and Kerala [ARIMA (5, 2, 3) with drift] show significant results for the data set. To stabilize the residual variance, logarithmic transformation followed by square transformation (for Andhra Pradesh and Kerala), cube root transformation (for Maharashtra), square root transformation (for Tamil Nadu) and fourth root transformation (for Karnataka) were used.

**Table 6.** Results of the 15-days-ahead forecasts (in millions) from 21 December 2020 to 4 January 2021 for five states (Maharashtra [MH], Karnataka [KA], Andhra Pradesh [AP], Tamil Nadu [TN] and Kerala [KL]).

Date	MH	KAR	AP	TN	KL
21 December 2020	1.90	0.91	0.88	0.81	0.71
22 December 2020	1.90	0.91	0.88	0.81	0.72
23 December 2020	1.91	0.91	0.88	0.81	0.72
24 December 2020	1.91	0.91	0.88	0.81	0.73
25 December 2020	1.91	0.92	0.88	0.81	0.73
26 December 2020	1.92	0.92	0.88	0.81	0.74
27 December 2020	1.92	0.92	0.88	0.81	0.75
28 December 2020	1.92	0.92	0.88	0.82	0.75
29 December 2020	1.93	0.92	0.88	0.82	0.76
30 December 2020	1.93	0.92	0.88	0.82	0.76
31 December 2020	1.93	0.92	0.88	0.82	0.77
1 January 2021	1.94	0.92	0.88	0.82	0.78
2 January 2021	1.94	0.92	0.88	0.82	0.78
3 January 2021	1.94	0.93	0.89	0.82	0.79
4 January 2021	1.94	0.93	0.89	0.82	0.80

### Comparative study

#### Influence on the forecast after changing the time series length

In order to study the performance of each model for the varied time series, i.e. after eliminating the days with zero reported cases, we used MAPE and RMSE for the ARIMA model, since it was the best-fitting model. The comparison is shown in Table 7. It can be seen that the MAPE and RMSE values provided by a full-length time series are less as compared with the truncated time series. Hence we used the full-length time series data using the ARIMA model for forecasting in India and its five states.

**Table 7.** Results of the comparison between the full-length time series and truncated time series.

	Infected		Deceased	
Measure	Full-length time series	Truncated time series	Full-length time series	Truncated time series
RMSE	0.20	2.03	0.22	2.24
MAPE	0.03	0.05	2.99	0.07

#### Implications of the findings and policy recommendations

Considering the present situation in India, Internet of Things–based smart disease surveillance systems have the potential to be a major breakthrough in efforts to control the current pandemic. With much of the infrastructure already in place (i.e. smartphones, wearable technologies, internet access), the role this technology can play in limiting the spread of the pandemic involves only the collection and analysis of data.^24^ Another use can be in understanding the characteristics of spatiotemporal clustering of the COVID-19 epidemic, as *R*_0_ is critical in effectively preventing and controlling the pandemic.^25^

#### Limitations and intervention scenarios

COVID-19 has been affected by a number of factors. Some studies have revealed how multiple variables contribute to the spread of the virus,^26^ but with the inclusion of proper interventions, the spread of COVID-19 can be monitored.^27^ However, it should be mentioned that this forecast is strongly related to the past pattern. The current situation in India represents a declining trend in daily reported infections. Our aim through this article is to compare the considered models in forecasting this pandemic based on the data set that is used. Also, considering the fact that there might have been a greater number of infections and deaths in the country as compared with what is being reported, this study is limited to the cases that have been reported. Simulations are beyond the of scope for this article.

## Conclusions

In this article we adopted the exponential, logistic, Gompertz and ARIMA models for short-term forecasting of the COVID-19 outbreak in India and its five most affected states. The results of all the considered methods show that the cumulative number of infected cases and deaths due to COVID-19 are increasing day by day in India and its most affected states. As per the prediction, there will be around 3.42 million additional infected cases and about 0.006 million new deaths will be reported in India in the 15 days from 21 December 2020 to 4 January 2021. Among the four models, we found that the ARIMA model provided a better fit and gave a more reliable forecast using epidemiological data for India. After the announcement of various unlock phases, Maharashtra remains a highly affected state in India due to COVID-19. An increase in the number of infected cases is directly related to an increase in the number of testing facilities and the interstate movement of people. Through updating these data and applying the models at the regional level, some valuable and far more accurate predictions can be obtained.

## Supplementary Material

ihab031_Supplemental_File

## Data Availability

None.
